# Impact of Clinicians' Use of Electronic Knowledge Resources on Clinical and Learning Outcomes: Systematic Review and Meta-Analysis

**DOI:** 10.2196/13315

**Published:** 2019-07-25

**Authors:** Lauren A Maggio, Christopher A Aakre, Guilherme Del Fiol, Jane Shellum, David A Cook

**Affiliations:** 1 Department of Medicine Uniformed Services University of the Health Sciences Bethesda, MD United States; 2 Division of General Internal Medicine Mayo Clinic College of Medicine and Science Rochester, MN United States; 3 Department of Biomedical Informatics University of Utah School of Medicine Salt Lake City, UT United States; 4 Center for Translational Informatics and Knowledge Management Mayo Clinic Rochester, MN United States

**Keywords:** medical education, information systems, educational technology, clinical decision support, health information technology

## Abstract

**Background:**

Clinicians use electronic knowledge resources, such as Micromedex, UpToDate, and Wikipedia, to deliver evidence-based care and engage in point-of-care learning. Despite this use in clinical practice, their impact on patient care and learning outcomes is incompletely understood. A comprehensive synthesis of available evidence regarding the effectiveness of electronic knowledge resources would guide clinicians, health care system administrators, medical educators, and informaticians in making evidence-based decisions about their purchase, implementation, and use.

**Objective:**

The aim of this review is to quantify the impact of electronic knowledge resources on clinical and learning outcomes.

**Methods:**

We searched MEDLINE, Embase, PsycINFO, and the Cochrane Library for articles published from 1991 to 2017. Two authors independently screened studies for inclusion and extracted outcomes related to knowledge, skills, attitudes, behaviors, patient effects, and cost. We used random-effects meta-analysis to pool standardized mean differences (SMDs) across studies.

**Results:**

Of 10,811 studies screened, we identified 25 eligible studies published between 2003 and 2016. A total of 5 studies were randomized trials, 22 involved physicians in practice or training, and 10 reported potential conflicts of interest. A total of 15 studies compared electronic knowledge resources with no intervention. Of these, 7 reported clinician behaviors, with a pooled SMD of 0.47 (95% CI 0.27 to 0.67; *P*<.001), and 8 reported objective patient effects with a pooled SMD of 0.19 (95% CI 0.07 to 0.32; *P*=.003). Heterogeneity was large (I^2^>50%) across studies. When compared with other resources—7 studies, not amenable to meta-analytic pooling—the use of electronic knowledge resources was associated with increased frequency of answering questions and perceived benefits on patient care, with variable impact on time to find an answer. A total of 2 studies compared different implementations of the same electronic knowledge resource.

**Conclusions:**

Use of electronic knowledge resources is associated with a positive impact on clinician behaviors and patient effects. We found statistically significant associations between the use of electronic knowledge resources and improved clinician behaviors and patient effects. When compared with other resources, the use of electronic knowledge resources was associated with increased success in answering clinical questions, with variable impact on speed. Comparisons of different implementation strategies of the same electronic knowledge resource suggest that there are benefits from allowing clinicians to choose to access the resource, versus automated display of resource information, and from integrating patient-specific information. A total of 4 studies compared different commercial electronic knowledge resources, with variable results. Resource implementation strategies can significantly influence outcomes but few studies have examined such factors.

## Introduction

Clinicians and trainees frequently identify clinical questions while caring for patients [1]. They have been trained, and often attempt, to answer these questions using a variety of resources, including increasing use of electronic resources [2-4]. Electronic knowledge resources have been defined as “electronic (computer-based) resources comprising distilled (synthesized) or curated information that allows clinicians to select content germane to a specific patient to facilitate medical decision making” [5]. Commonly used electronic knowledge resources include commercial products, such as UpToDate, Micromedex, and Epocrates [6,7]; locally developed products, such as McMaster Premium LiteratUre Service (PLUS) [8]; and crowdsourced resources, such as Wikipedia [9]. Electronic knowledge resources are related to, but distinct from, decision-support tools that provide pop-up alerts, reminders, and other push notifications or databases of unsynthesized information, such as MEDLINE.

Electronic knowledge resources are commonly used in clinical practice and typically require significant resources, including the financial investment in procuring access and clinicians' investment of time in learning to use them [10]. However, their impact on patient care and learning outcomes is incompletely understood [4,11]. Previous reviews of health information resources have, in general, broadly focused on clinical decision-support tools [12,13]. One review characterized features of clinical information retrieval technology that promote its use [14] but did not examine the specific knowledge resources themselves. Another review of clinicians' information-seeking behaviors identified textbooks, colleagues, journal articles, professional websites, and medical libraries as information sources but did not report the outcomes associated with using these sources [15]. A review of clinical questions noted the use of knowledge resources to answer such questions but did not directly address knowledge resources [1]. Moreover, the age of these reviews (ie, the most recent having been published in 2014) limits their application to current practice. An up-to-date, comprehensive synthesis of evidence regarding the effectiveness of electronic knowledge resources could guide clinicians, health care system administrators, medical educators, and informaticians in making evidence-based decisions about their purchase, implementation, and use. Thus, we conducted a systematic review to answer the following question: What is the impact of electronic knowledge resources for clinicians on clinical and learning outcomes?

## Methods

This study is part of a large systematic review of knowledge resources and point-of-care learning that was planned, conducted, and reported in adherence to standards of quality for reporting meta-analyses [16].

### Search Strategy and Study Selection

With support from an experienced reference librarian, on February 14, 2017, we simultaneously searched MEDLINE, Embase, PsycINFO, and the Cochrane Library Database using Ovid’s integrated search interface for comparative studies of electronic knowledge resources. We used the databases’ controlled vocabulary thesauri, Web searches, the research teams' files, and previous reviews [1,6,13,14,17] to create and refine the search strategy and supplemented the database search by examining the full bibliography of these reviews. Search terms included a combination of keywords and controlled vocabulary terms (eg, *information-seeking behavior*, *point-of-care systems*, *drug information services*, *UpToDate*, and *Micromedex*). Multimedia Appendix 1 describes the complete search strategy. We limited our search to studies published after January 1, 1991, the year in which the World Wide Web was first described. We made no exclusions based on language.

### Article Selection

We included all original, comparative studies that evaluated clinicians' use of an electronic knowledge resource, using quantitative outcomes of knowledge, skills in a test setting, attitudes, behaviors with real patients, patient effects, and costs. We required that outcome measures relate to a clinical decision for a specific patient or clinical vignette; we excluded studies measuring only general experiences or overall perceived impact. Measurements in a test setting had to be objectively assessed, as opposed to clinician-reported, and performed without immediate support from the knowledge resource (ie, evaluating sustained impact on knowledge after a period of access, rather than concurrent decision support). Measurements in the care of real patients could be clinician-reported (eg, “found an answer”) or objectively assessed and could reflect concurrent support or sustained impact.

We defined *electronic knowledge resource* as quoted in the Introduction, which was adapted from the definition proposed by Lobach [12].We defined clinicians as practitioners or students in a health-related field with direct responsibility for patient-related decisions; this included, but was not limited to, physicians, nurse practitioners, physician assistants, certified nurse anesthetists, pharmacists, midwives, dentists, and psychologists. We excluded studies focused solely on nurses and allied health professionals. We included studies making a comparison with a separate intervention, including randomized, nonrandomized, and crossover designs, or with baseline performance (ie, single-group, pre-/postintervention studies).

Reviewers (DAC, CAA, and LAM) worked independently and in duplicate to screen each identified study for inclusion, first reviewing the title and abstract and then reviewing the full text if needed; the kappa indicating interrater reliability should be greater than or equal to .70. All disagreements were resolved by consensus.

### Data Abstraction

Two reviewers (DAC and LAM) used a standardized abstraction form to independently extract data from all included studies, resolving all disagreements by consensus. We extracted information about the participants, topic, resources used, outcomes, and potential conflicts of interest. We appraised study quality using the Newcastle-Ottawa Scale as modified for education [18,19], which evaluates sample selection and comparability, blinding of assessment, and attrition. We converted all quantitative results, including odds ratios (ORs) [20], to standardized mean differences (SMDs).

### Data Synthesis

We conducted a meta-analysis to pool SMDs whenever three or more studies shared conceptually aligned, between-intervention contrasts [20]. In accordance with our study protocol, we used random-effects meta-analysis because we anticipated pooling across different resources, with likely different effects. We planned to weight studies by the number of users, but most studies reporting clinical outcomes reported only the number of patients or hospitals. Thus, we weighted analyses of knowledge and skills outcomes by the number of users, and we weighted analyses of clinician behaviors and patient effects by the number of patients, with exceptions as noted in the text. We conducted sensitivity analyses limited to randomized trials, recent publications (ie, after 2007), and studies of physicians in practice or postgraduate trainees. We planned to check for publication bias using funnel plots, but the small number of studies precluded meaningful analysis. We estimated heterogeneity using I^2^.

For studies that did not permit meta-analysis, we synthesized results using narrative methods, taking into account key differences in study design, study quality, intervention, and context.

## Results

**Figure 1:**
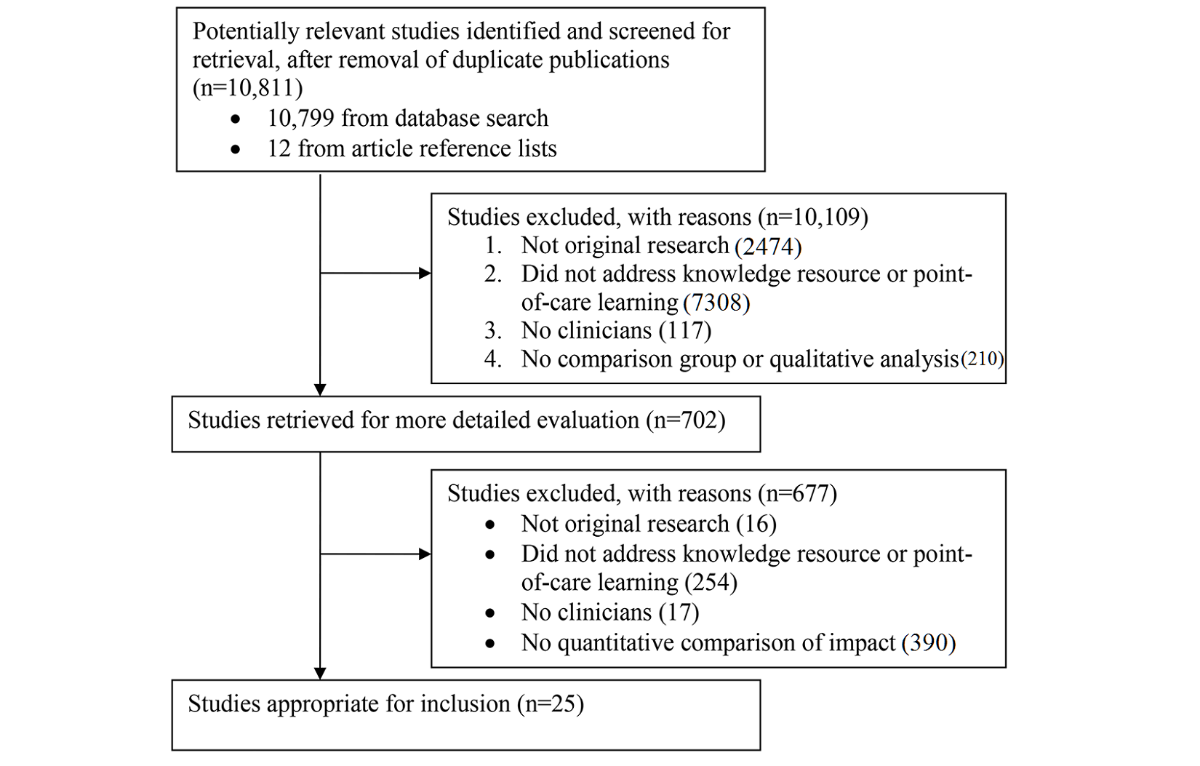
Trial flowchart.

We identified 10,811 potentially relevant studies: 10,799 studies in our literature search and 12 from our examination of previous reviews. From these, we included 25 comparative studies evaluating the impact of electronic knowledge resources (see Figure 1) [21-45].

### Study Characteristics

Table 1 summarizes study characteristics and Table 2 provides detailed information about each study. Out of 25 studies, 20 (80%) investigated electronic knowledge resources in the context of patient care, while 5 (20%) took place in laboratory or test settings. Nearly all studies (22/25, 88%) included physicians in practice or in training. Other studies included nurse practitioners or mixed user groups. Common topics included general medicine (15/25, 60%), surgery (5/25, 20%), and pediatrics (5/25, 20%). All studies were published between 2003 and 2016 and were in English.

**Table 1:** Summary of key study characteristics and quality.

Study characteristic			Studies (N=25), n (%)
**Participants^a^**			
	Practicing physicians		12 (48)
	Physicians in postgraduate training		12 (48)
	Medical students		4 (16)
	Nurse practitioners		3 (12)
	Mix of user groups		7 (28)
**Clinical topics^a^**			
	General medicine		15 (60)
	Pediatrics		5 (20)
	Surgery		5 (20)
	Emergency medicine		3 (12)
	Medical specialties		3 (12)
	Anesthesia		2 (8)
	Laboratory medicine, pathology, and radiology		1 (4)
	Pharmacy		1 (4)
**Patient setting**			
	Outpatient		11 (44)
	Inpatient		7 (28)
	Unspecified		3 (12)
**Electronic knowledge resources^b^**			
	UpToDate		6 (24)
	InfoRetriever		5 (20)
	Clinical evidence		2 (8)
	DynaMed		2 (8)
	Epocrates		2 (8)
	MD Consult		2 (8)
	Micromedex		2 (8)
	Trip^c^		2 (8)
	Other		9 (36)
**Comparison resources^b^**			
	MEDLINE		4 (16)
	User choice of any nonknowledge resource		3 (12)
	Journals		3 (12)
	Paper resources		3 (12)
	Curated (eg, library subject guides)		3 (12)
	Google		2 (8)
	Other search engines		2 (8)
**Quality measures**			
	**Number of groups**		
		One study group	5 (20)
		Crossover design	9 (36)
		Two or more study groups	11 (44)
	**Newcastle-Ottawa Scale results**		
		Score: ≥4	8 (32)
		Representative: yes	9 (36)
		Selection of comparison group: same community	9/12 (75)^d^
		Comparability of comparison group: high	5/12 (42)^d^
		Follow-up: high (>75%)	16 (64)
		Blinded outcomes assessment: yes	9 (36)
**Funding**			
	Potential conflict of interest		10 (40)

^a^The number of studies in some subgroups may add up to more than the total number of studies, and percentages may be more than 100%, because several studies included combinations of clinicians and/or study topics.

^b^Selected list of electronic knowledge resources and comparison resources; other resources were studied with lower frequency.

^c^Trip: Turning Research Into Practice.

^d^Percentage of two-group studies.

**Table 2:** Detailed information about each study.

Author, year	User type	Topic	Knowledge resource	Comparison	Outcomes
Leung, 2003 [21]	Medical students	General medical and surgery	InfoRetriever	NI^a^ and ORes^b^	Attitudes
Schwartz, 2003 [22]	Practicing physicians	General medical	Clinical evidence, DynaMed, InfoRetriever, and Trip^c^	ORes	Knowledge and skills
D'Alessandro, 2004 [23]	Practicing physicians and residents	Pediatrics	MD Consult and Micromedex	ORes and KR^d^	Knowledge and skills
Alper, 2005 [24]	Practicing physicians and nurse practitioners	General medical and pediatrics	DynaMed, InfoRetriever, Medscape, MD Consult, and UpToDate	ORes	Knowledge and skills
Grad, 2005 [25]	Residents	General medical	InfoRetriever	NI	Knowledge and skills
Grad, 2005 [26]	Residents	General medical	InfoRetriever	KR	Knowledge and skills
Greiver, 2005 [27]	Practicing physicians	General medical	Angina software^e^	NI	Behaviors and patient effect
Bochicchio, 2006 [28]	Residents	General medical, surgery, anesthesia, and emergency medicine	Johns Hopkins Antibiotics Guide^e^	NI	Knowledge and skills
Maviglia, 2006 [29]	Practicing physicians, residents, and nurse practitioners	General medical and medical specialties	Micromedex	KR	Knowledge and skills
Ramnarayan, 2006 [30]	Residents	Pediatrics	Isabel	NI	Behaviors
Rudkin, 2006 [31]	Practicing physicians and residents	Emergency medicine	Epocrates, Tarascon Pharmacopeia, The 5-Minute Clinical Consult, and qID	ORes	Knowledge and skills
Emery, 2007 [32]	Residents and nurse practitioners	General medical	GRAIDS^e,f^	NI	Behaviors and patient effect
King, 2007 [33]	Residents and medical students	Anesthesia and pediatrics	Clinical evidence module^e^	NI	Behaviors and patient effect
Magrabi, 2007 [34]	Practicing physicians	General medical	MIMS^g^ and Quick Clinical	NI	Attitudes
Skeate, 2007 [35]	Residents and medical students	Laboratory medicine, pathology, and radiology	Report Support^e^	NI	Knowledge and skills
Van Duppen, 2007 [36]	Practicing physicians and residents	General medical	Clinical evidence and Trip	ORes and KR	Knowledge and skills
Bonis, 2008 [37]	Mixed users^h^	Mixed topics	UpToDate	NI	Behaviors and patient effect
Hoogendam, 2008 [38]	Practicing physicians and residents	General medical	UpToDate	ORes	Knowledge and skills
Lyman, 2008 [39]	Practicing physicians	Pharmacy	Epocrates	NI	Behaviors
Isaac, 2012 [40]	Mixed users	General medical and surgery	UpToDate	NI	Behaviors and patient effect
Reed, 2012 [41]	Practicing physicians	General medical	PIER^i^ and UpToDate	NI and KR	Knowledge and skills
Kuhn, 2015 [42]	Mixed users	Medical specialties	eAAP^e,j^	NI	Patient effect
Chow, 2016 [43]	Mixed users	General medical, medical specialties, pharmacy, surgery, and mixed topics	ARUSC^e,k^	KR	Behaviors
Luther, 2016 [44]	Practicing physicians	Emergency medicine, pediatrics, and surgery	SCAMP^e,l^	NI	Behaviors, patient effect, and costs
Saparova, 2016 [45]	Medical students	Mixed topics	AccessMedicine, UpToDate, and Wikipedia	KR	Knowledge and skills

^a^NI: Knowledge resource compared versus no intervention.

^b^ORes: Knowledge resource compared versus other resource.

^c^Trip: Turning Research Into Practice.

^d^KR: Comparison between knowledge resources.

^e^Denotes a locally developed resource.

^f^GRAIDS: Genetic Risk Assessment on the Internet with Decision Support.

^g^MIMS: Monthly Index of Medical Specialties.

^h^An undifferentiated mix of users.

^i^PIER: Physicians’ Information and Education Resource.

^j^eAAP: Emergency Asthma Action Plan.

^k^ARUSC: Antibiotic Utilization and Surveillance-Control.

^l^SCAMP: Standardized Clinical Assessment and Management Plans.

**Figure 2:**
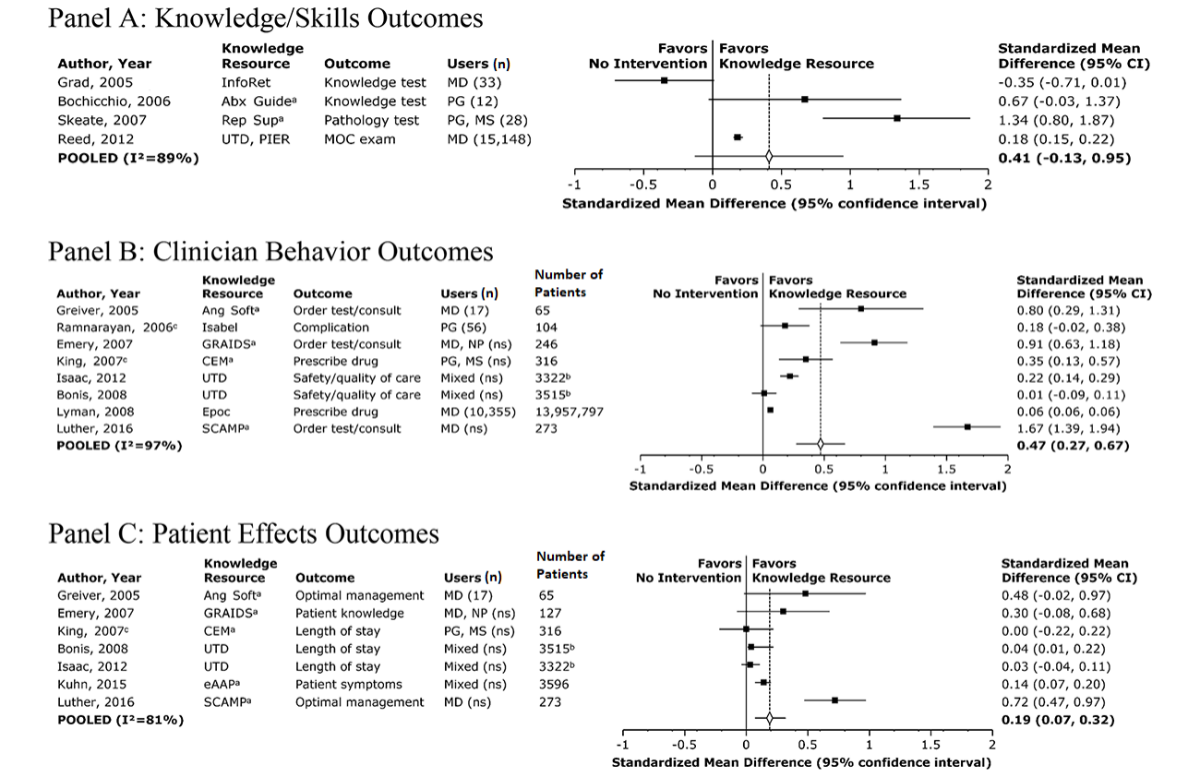
Comparative usage of electronic knowledge resources versus no intervention. Knowledge outcome analyses are weighted by user, while behavior and patient effects analyses are weighted by patients or hospitals. “a” denotes a locally developed resource; “b” is the number of hospitals, not patients; “c” indicates no comparison group (ie, one-group, pre-/postintervention study). Abx Guide: Johns Hopkins Antibiotic Guide; Ang Soft: angina software; CEM: clinical evidence module; eAAP: Emergency Asthma Action Plan; Epoc: Epocrates; GRAIDS: Genetic Risk Assessment on the Internet with Decision Support; InfoRet: InfoRetriever; MD: practicing physicians; MOC: Maintenance of Certification; MS: medical students; NP: nurse practitioners; ns: not specified; PG: residents; PIER: Physicians’ Information and Education Resource; Rep Sup: Report Support; SCAMP: Standardized Clinical Assessment and Management Plans; UTD: UpToDate.

**Figure 3:**
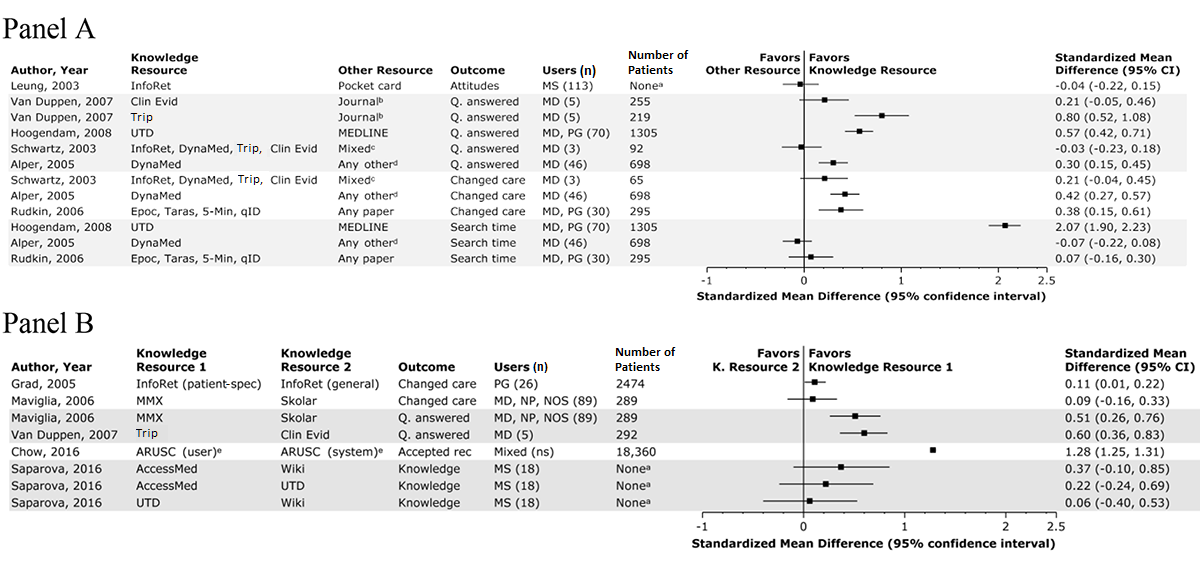
Impact of electronic knowledge resources in comparison with other resources (Panel A) and alternate electronic knowledge resources (Panel B). All analyses are weighted by patients except as noted. “a” refers to analysis weighted by users; “b” means the comparison group (ie, study data) is the same for these contrasts; “c” means the comparison type “Mixed” indicates a comparison with both electronic and nonelectronic knowledge resources; “d” means the comparison type “Any other” indicates users could select any resource, except the ones it was being compared against; “e” denotes a locally developed resource. 5-min: 5-Minute Clinical Consult; AccessMed: AccessMedicine; ARUSC: Antibiotic Utilization and Surveillance-Control; Clin Evid: clinical evidence; Epoc: Epocrates; InfoRet: InfoRetriever; K: Knowledge; MD: practicing physicians; MMX: Micromedex; MS: medical students; NOS: not otherwise specified; NP: nurse practitioners; ns: not specified; PG: residents; Q: question; rec: recommendation; spec: specific; Taras: Tarascon Pharmacopeia; Trip: Turning Research Into Practice; UTD: UpToDate; Wiki: Wikipedia.

The electronic knowledge resources most commonly evaluated were UpToDate (6/25, 24%) and InfoRetriever (5/25, 20%). Several studies evaluated more than one resource. The 25 studies reported 29 distinct contrasts. Out of 29, 15 contrasts (52%) compared electronic knowledge resources with no intervention; 7 (24%) compared electronic knowledge resources with resources not meeting our definition of electronic knowledge resources, such as MEDLINE or a paper resource, hereafter collectively labeled *other resources*; and 7 (24%) compared one electronic knowledge resource against another (eg, Micromedex vs SkolarMD or two implementations of the same resource, such as presentation as a desktop vs mobile version). Across the 29 contrasts, we extracted 48 discrete outcomes, reflecting knowledge and skills (24/48, 50%), behaviors in practice (10/48, 21%), patient effects (10/48, 21%), attitudes (3/48, 6%), and costs (1/48, 2%). Selected contrasts and outcomes are reported in Figures 2 and 3; Multimedia Appendix 2 lists all contrasts and outcomes.

### Study Quality

When reported, the number of enrolled users ranged from 3 to 15,148; 7 studies out of 25 (28%) did not report the number of users, and 4 (16%) did not report user demographics. A total of 11 (44%) of the 25 studies included two or more groups, of which 5 (45%) were randomized. Assessors were blinded to the study intervention in 9 (36%) of the 25 studies. The mean Newcastle-Ottawa Scale quality score (maximum 6 points) was 2.3 (SD 1.6). In 15 out of 25 studies (60%), outcomes were determined objectively (eg, based on patient records, computer logs, or test scores), including all studies that reported patient outcomes. The other 10 studies (40%) reported only clinician-reported measures (eg, “I found an answer”). Only 9 of 25 studies (36%) enrolled users that were considered representative of the larger community of potential participants. A total of 11 studies (44%) had a separate comparison group; among these, 9 (82%) drew the comparison group from the same community and 5 (45%) were randomized. A total of 16 out of 25 studies (64%) reported high participant follow-up. A total of 10 studies (40%) reported potential financial conflicts of interest (eg, industry grant, discounted or free product pricing, involvement of resource creators, or employment by industry). A total of 6 studies (24%) did not report funding sources (see Table 3).

### Synthesis: Comparisons With No Intervention

A total of 15 studies out of 25 (60%) compared one or more electronic knowledge resources with no intervention, including comparisons of usual practice without versus with access to the resource, reporting a total of 22 outcomes [21,25-28,30,32-35,37,39-42,44]. Of these 15 studies, 9 (60%) reported potential conflicts of interest.

Out of these 15 studies, 4 (27%) reported knowledge or skill outcomes, evaluating InfoRetriever, UpToDate, American College of Physicians (ACP) Physicians’ Information and Education Resource (PIER), and three local resources, alone or in varying combinations. The pooled SMD was 0.41 (95% CI –0.13 to 0.95; *P*=.14; see Figure 2, Panel A). Inconsistency was high, with individual SMDs ranging from –0.35 to 1.34 and an I^2^ of 89%. None of these studies were randomized and only 1 out of the 4 (25%) was published since 2007. Limiting this analysis to the 3 studies out of 4 (75%) without a potential conflict of interest yielded an SMD of 0.35 (95% CI –0.29 to 0.99; *P*=.29). Limiting the analysis to the 3 studies (75%) enrolling physicians in practice or postgraduate trainees revealed an SMD of 0.10 (95% CI –0.34 to 0.54; *P*=.65). Out of the 4 studies, 2 (50%) explored attitudes about information seeking and evidence-based medicine, with results showing improved, neutral, and worsened attitudes, depending on the attitude statement, after use of knowledge resources [21,34].

**Table 3:** Quality appraisal of included studies.

Author, year	Users, n	Study design	Newcastle-Ottawa Scale score^a^	Representativeness^b^	Comparable cohorts^c^	Follow-up^d^	Objective outcomes^e^	Blinded^f^	COI^g^
Leung, 2003 [21]	113	1 group, crossover	1	Yes	N/A^h^	Low	Yes	No	Yes
Schwartz, 2003 [22]	3	1 group, crossover	1	No	N/A	High	No	No	No
D'Alessandro, 2004 [23]	52	1 group, crossover	1	No	N/A	High	No	No	No
Alper, 2005 [24]	82	1 group, crossover	1	No	N/A	High	No	No	Yes
Grad, 2005 [25]	37	≥2 groups	4	Yes	Similar	High	Yes	No	No
Grad, 2005 [26]	26	1 group, crossover	0	No	N/A	Low	No	No	No
Greiver, 2005 [27]	18	≥2 groups, RCT^i^	4	No	Similar	High	Yes	No	No
Bochicchio, 2006 [28]	12	≥2 groups, RCT	4	No	Similar	High	Yes	Yes	Yes
Maviglia, 2006 [29]	279	≥2 groups, RCT	3	No	Similar	Low	No	No	Yes
Ramnarayan, 2006 [30]	80	1 group	2	Yes	N/A	Low	Yes	Yes	Yes
Rudkin, 2006 [31]	30	1 group, crossover	1	No	N/A	High	No	No	No
Emery, 2007 [32]	Not specified	≥2 groups, RCT	4	Yes	Similar	Low	Yes	Yes	Yes
King, 2007 [33]	Not specified	1 group	0	No	N/A	Low	Yes	No	No
Magrabi, 2007 [34]	227	1 group	0	No	N/A	Low	No	No	Yes
Skeate, 2007 [35]	30	≥2 groups, RCT	3	No	Similar	High	Yes	No	No
Van Duppen, 2007 [36]	5	1 group, crossover	1	No	N/A	High	No	No	No
Bonis, 2008 [37]	Not specified	≥2 groups	4	No	N/A	High	Yes	Yes	Yes
Hoogendam, 2008 [38]	70	1 group, crossover	1	No	N/A	High	No	No	No
Lyman, 2008 [39]	10,355	≥2 groups	4	Yes	Similar	High	Yes	Yes	Yes
Isaac, 2012 [40]	Not specified	≥2 groups	4	No	N/A	High	Yes	Yes	Yes
Reed, 2012 [41]	15,148	≥2 groups	6	Yes	Similar	High	Yes	Yes	No
Kuhn, 2015 [42]	Not specified	1 group	1	Yes	N/A	Low	Yes	No	No
Chow, 2016 [43]	Not specified	1 group, crossover	2	Yes	N/A	Low	No	Yes	No
Luther, 2016 [44]	Not specified	1 group	2	Yes	N/A	High	Yes	No	No
Saparova, 2016 [45]	18	≥2 groups	3	No	Similar	High	Yes	Yes	No

^a^The score for this scale can be a maximum of 6 points.

^b^“Yes” indicates that the study is truly representative of the average clinician in the community, while “No” indicates that it is not.

^c^“Similar” indicates that the comparison group was drawn from the same community.

^d^“High” indicates that participant follow-up was ≥75%; “Low” indicates that follow-up was <75% or unclear.

^e^“Yes” indicates that at least one outcome was determined objectively; “No” indicates outcomes were not determined objectively.

^f^“Yes” indicates blinded outcomes; “No” indicates no blinding.

^g^“No” indicates no conflict of interest (COI) reported or identified by the reviewer team; “Yes” indicates a reported or identified potential COI.

^h^N/A: not applicable (ie, no separate comparison group).

^i^RCT: randomized controlled trial.

Out of the 15 studies, 8 (53%) reported behavior outcomes, such as appropriate therapy recommendations and test orders, evaluating combinations of Epocrates, Isabel, UpToDate, and four local resources [27,30,32,33,39,40,44]. The pooled SMD was 0.47 (95% CI 0.27 to 0.67; *P*<.001; see Figure 2, Panel B). Inconsistency was again high, with individual SMDs ranging from 0.01 to 1.67 and an I^2^ of 97%. Out of the 8 studies, 2 (25%) were randomized [27,32]. Limiting analyses to the 4 studies (50%) published since 2007 revealed similar results, with an SMD of 0.41 (95% CI 0.10 to 0.71; *P*=.01). Alternately, limiting to the 3 studies (38%) without a potential conflict of interest yielded an SMD of 0.94 (95% CI 0.02 to 1.86; *P*=.05). Lastly, limiting analysis to the 7 studies (88%) that included physicians in practice or postgraduate trainees produced an SMD of 0.49 (95% CI 0.27 to 0.70; *P*<.001).

Out of the 15 studies, 7 (47%) reported patient effects, including complications, length of stay, optimal management, and mortality, evaluating UpToDate and five locally developed resources [27,32,33,37,40,42,44]. Pooling nonmortality outcomes across these 7 studies revealed an SMD of 0.19 (95% CI 0.07 to 0.32; *P*=.003; see Figure 2, Panel C). Inconsistency was again high, with individual SMDs ranging from 0 to 0.72 and an I^2^ of 81%. Out of these 7 studies, 2 (29%) were randomized [26,31]. Limiting analyses to the 4 studies out of 7 (57%) published since 2007 revealed a similar SMD of 0.20 (95% CI 0.05 to 0.35; *P*=.01). Limiting to the 4 studies out of 7 (57%) without potential conflicts of interest yielded an SMD of 0.31 (95% CI 0.01 to 0.61; *P*=.04). Focusing on the 6 studies out of 7 (86%) that enrolled physicians in practice or postgraduate trainees produced an SMD of 0.22 (95% CI 0.09 to 0.36; *P*=.001). The 2 studies out of 7 (29%) reporting mortality outcomes, both funded by UpToDate, compared hospitals that did versus did not have access to UpToDate. Out of these 2 studies, 1 (50%) found a very small but statistically significant association between the use of UpToDate and lower mortality (absolute risk difference –0.1%; N=3322 hospitals) [40]; the other found no statistically significant association (risk-adjusted z-score 0.18; N=5515 hospitals) [37].

Out of the 15 studies, 1 (7%) objectively evaluated cost reductions associated with implementation of a local resource; this study found a statistically significant 49% reduction in the cost of care (95% CI 0.46 to 0.53) compared with preimplementation [44].

### Synthesis: Comparisons With Other Resources

A total of 7 studies out of 25 (28%) compared electronic knowledge resources with other information resources that were provided instead of the knowledge resource and that did not meet our definition of electronic knowledge resources (see Figure 3, Panel A) [21-24,31,36,38]. Variation in comparisons and outcomes precluded meaningful meta-analysis. Out of the 7 studies, 2 (29%) found mixed results for the use of electronic knowledge resources on personal digital assistants (PDAs) compared with paper resources. In 1 crossover study (50%), residents given a PDA with electronic knowledge resources (eg, Epocrates and Tarascon Pharmacopeia) demonstrated improvements in self-reported patient management (SMD 0.38, 30 users, 295 patients), compared with resource access limited to print materials [30]. The second study, conducted by the creators of InfoRetriever, found essentially no difference in attitudes about evidence-based medicine when comparing use of a PDA preloaded with InfoRetriever versus an evidence-based medicine pocket card (SMD –0.04, 113 users) [21].

Out of the 7 studies, 2 (29%) suggested that clinicians found answers to more questions, and more rapidly, when using electronic knowledge resources than when using journal-based resources. Out of these 2 studies, 1 crossover study (50%) compared general practitioners’ use of Turning Research Into Practice (Trip) and clinical evidence with their use of journal articles from the BMJ and found that these electronic knowledge resources were associated with more frequently finding answers (Trip vs the BMJ: SMD 0.80, 5 users, 219 patients; clinical evidence vs the BMJ: SMD 0.21, 5 users, 255 patients) [36]. Another study (1/2, 50%) reported a statistically significant association between the use of UpToDate and answering more questions (SMD 0.57, 70 users, 1305 patients) and finding answers more quickly (SMD 2.07), in comparison with clinicians using PubMed [38].

Out of the 7 studies, 3 (43%) examined electronic knowledge resources in comparison with a user’s choice of any other information resources (eg, Google and textbooks) and reported mixed findings. In a randomized crossover study (1/3, 33%) authored by the founder of DynaMed, physicians using DynaMed reported that they found answers more often (SMD 0.30, 46 users, 698 patients) and that answers more often changed patient care (SMD 0.42), although finding answers took slightly, but not statistically significantly, longer (SMD –0.07) [24]. Another study (1/3, 33%) compared the use of InfoRetriever, DynaMed, Trip, and clinical evidence against a user’s choice of any other resources; the study found that the use of these electronic knowledge resources was not significantly associated with clinician-reported success in answering questions (SMD –0.03, 3 users, 92 patients) or changes in care (SMD 0.21, 3 users, 65 patients) [22]. In a third study (1/3, 33%), which is not represented in Figure 3, Panel A, because of insufficient extractable data, pediatricians were randomized to use an online pediatrics library or a resource of their choice and found no statistically significant difference in questions answered or changes in care [23].

### Synthesis: Comparisons Between Electronic Knowledge Resources

The high inconsistency noted in the meta-analyses above may suggest substantial differences between knowledge resources in their implementation (eg, training, policies, and technical support to encourage or facilitate use) and design. Studies comparing different electronic knowledge resources, designs, or implementation strategies can help identify best practices. We identified 7 such studies out of 25 (28%; see Figure 3, Panel B) [23,26,29,36,41,43,45].

Out of these 7 studies, 2 (29%) reported associations between different resource implementation strategies of the same knowledge resource and changes in care. In 1 study (50%), clinicians who were allowed to optionally use a local electronic knowledge resource more often followed the resource’s suggestion on antibiotic use compared with when they were provided such information without their request (SMD 1.28, 18,360 patients) [43]. The other study compared two subsections of InfoRetriever: one that employed user-entered patient data to provide patient-specific information and recommendations and the other containing general information resources, such as The 5-Minute Clinical Consult, Cochrane Reviews, Information Patient-Oriented Evidence that Matters (Info-POEMs), and guideline summaries. This crossover study determined that the patient-specific resources were associated with a slight but statistically significant improvement in clinician-reported changes in care (SMD 0.11, 26 users, 2474 patients) [26].

Out of the 7 studies, 4 (57%) focused on head-to-head comparisons of different electronic knowledge resources. Out of these 4 studies, 1 crossover study (25%) found no statistically significant difference on a knowledge test for 18 medical students who had used Wikipedia, AccessMedicine, or UpToDate (UpToDate vs Wikipedia SMD 0.06; AccessMedicine vs UpToDate SMD 0.22; AccessMedicine vs Wikipedia SMD 0.37) [45]. Another randomized study (1/4, 25%) found no statistically significant differences between Micromedex and SkolarMD in the frequency of answering questions (SMD 0.51, 89 users, 289 patients) or clinician-reported changes in patient care (SMD 0.09) [29]. A randomized crossover study (1/4, 25%) found that clinicians could answer questions more often when using Trip than when using clinical evidence (SMD 0.60, 5 users, 292 patients) [36]. Finally, 1 study (25%) found a statistically significant difference in maintenance of certification exam scores between physicians using two electronic knowledge resources over an extended period; however, due to deliberately blinded reporting, it is not possible to know which resource (ie, PIER or UpToDate) was superior [41]. The effects of these resources in comparison with no intervention were reported earlier in this review.

## Discussion

### Principal Findings

We identified 25 studies that investigated the impact of electronic knowledge resources on patient and clinician outcomes and found results that are mixed and at times contradictory. Nevertheless, we found statistically significant associations between the use of electronic knowledge resources and improved clinician behaviors and patient effects. When compared with other resources, use of electronic knowledge resources was associated with increased success in answering clinical questions, with variable impact on speed. Comparisons of different implementation strategies of the same electronic knowledge resource suggest benefits from allowing clinicians to choose to access the resource, versus automated display of resource information, and from integrating patient-specific information. A total of 4 studies compared different commercial electronic knowledge resources, with variable results.

### Comparison With Other Reviews and Meta-Analyses

Clinicians frequently face clinical questions [1,46], which they are taught and expected to answer using some form of knowledge resources. Previous reviews have focused on interventions to promote knowledge resource adoption [14] or addressed knowledge resources as only one of many information technology tools [12,47]. This review expands upon prior work by focusing specifically on electronic knowledge resources and quantitatively estimating their impact on clinical outcomes and point-of-care learning. Our finding of limited evidence regarding different approaches to electronic knowledge resource implementation strategies parallels the paucity of evidence found in a previous review of health information technology [48].

### Limitations

As with all systematic reviews, our findings are constrained by the quality and quantity of published evidence. For example, only 6 studies reported patient effects and 5 were randomized. Inconsistency was high in all analyses. Additionally, lack of conceptual alignment precluded meta-analysis for comparisons of electronic knowledge resources with other resources or with different implementation strategies. Several studies allowed users access to multiple resources simultaneously, making interpretation difficult. Vague and incomplete reporting limited our ability to extract key information on study design, outcomes, contextual details (eg, setting and disease acuity), and resource design and implementation (eg, how participants accessed the resource, password requirements, or optimization for use on a mobile device) for several studies. A total of 10 studies presented potential conflicts of interest, which could bias results. However, sensitivity analyses limited to recent studies and studies without conflicts of interest generally yielded similar results. The small number of studies precluded meaningful evaluation of publication bias. We did not attempt to distinguish resources based on the developer's intended purpose (eg, education, decision support, or information) but instead focused on the resource's function and application (ie, decision making for a specific patient). Several studies are over a decade old, which limits their relevance to current resource implementations. We conducted our literature search in 2017, and studies published since that date were thus omitted from our analyses. This review has several strengths, including a comprehensive search of multiple databases by information professionals, duplicate review at all stages of screening and data extraction, and broad inclusion criteria encompassing a range of health professionals and topics.

### Implications

In this meta-analysis, use of electronic knowledge resources appeared to improve patient care and their continued use in clinical practice appears to be warranted. More specifically, these resources provide answers to clinician-initiated questions “just in time,” thus preserving clinicians' autonomy and workflow. This functionality contrasts with that of interruptive clinical decision-support systems, such as reminders and alerts, that have been associated with workflow disruption, alert fatigue, inappropriate recommendations, and provider dissatisfaction [49-51]. Use of electronic knowledge resources is also associated with enhanced durable learning (ie, improved performance on knowledge tests conducted without concurrent resource use). Clinicians may benefit from increased and more strategic use of electronic knowledge resources at various stages in training. Knowledge resources may be particularly important for practicing clinicians as part of their lifelong point-of-care learning activities [52]. The optimal promotion of durable learning may require resource features, such as spaced repetition and quizzing [53,54], that differ from those required for concurrent decision support (ie, maximal efficiency). Electronic knowledge resources offer flexibility allowing such features to be built in yet activated only for relevant learners and contexts.

The impact of electronic knowledge resources, while generally favorable, varied widely across studies. Such differences likely arise from specifics of the topic, clinical context, clinician specialty, and clinician stage of training, in addition to the knowledge resource itself. It seems unlikely that any one resource will optimally address all information needs; rather, health care organizations will likely need to make multiple electronic knowledge resources available and effectively integrate electronic knowledge resources into clinician workflows. Suboptimal integration results in suboptimal outcomes, as was seen in one study in this review [43]. Information tools, such as easily accessible online portals (eg, infobuttons [17]), might further help clinicians select resources appropriate for their specific questions and contexts.

Our review highlights several areas for improvement in the quality of research methods and reporting. For example, several studies failed to report the number of participants or participant demographics. Many studies did not use comparison groups, reported limited participant follow-up, or enrolled participants that were not considered representative of the larger community of potential participants. Also, 10 studies presented potential conflicts of interest (eg, funding from a resource vendor). Finally, the majority of studies lacked details on the design and implementation of the resources under investigation, and information was rarely reported regarding the cost—monetary and nonmonetary—of implementation, use, and maintenance. When planning future studies, researchers should consider and seek to mitigate these and other limitations.

Many uncertainties remain regarding optimal design, implementation strategies, and use of electronic knowledge resources. Unfortunately, studies comparing different knowledge resources or making comparisons with no intervention have largely failed to produce generalizable insights in this regard. Additional research is needed to clarify what works, in what context (ie, question type, topic, and clinical setting), and for what outcome. We believe that head-to-head studies of different resources, or different implementation strategies of a given resource, can provide such evidence; however, such studies must be guided by conceptual models and theories (eg, models and theories of information science and translational informatics). Noncomparative studies examining fundamental questions about information seeking, human factors, and user experience will also be useful. Outcomes of costs, both monetary and nonmonetary, will complement outcomes of effectiveness in supporting evidence-based decisions. Attention to these issues will permit more effective design, implementation strategies, and integration into the clinical workflow, which in turn will optimize electronic knowledge resources' benefits to patient care.

### Conclusions

Use of electronic knowledge resources is associated with a positive impact on clinician behaviors and patient effects. Further research into resource design and implementation strategies is needed.

## Appendix

Multimedia Appendix 1 Supplemental search strategies.

Multimedia Appendix 2 Detailed listing of all contrasts and outcomes by study.

## Abbreviations

ACP: American College of Physicians
ARUSC: Antibiotic Utilization and Surveillance-Control
COI: conflict of interest
eAAP: Emergency Asthma Action Plan
GRAIDS: Genetic Risk Assessment on the Internet with Decision Support
Info-POEMs: Information Patient-Oriented Evidence that Matters
KR: comparison between knowledge resources
MIMS: Monthly Index of Medical Specialties
NI: knowledge resource compared versus no intervention
OR: odds ratio
ORes: knowledge resource compared versus other resource
PDA: personal digital assistant
PIER: Physicians’ Information and Education Resource
PLUS: Premium LiteratUre Service
RCT: randomized controlled trial
SCAMP: Standardized Clinical Assessment and Management Plans
SMD: standardized mean difference
Trip: Turning Research Into Practice
