# Supplementary material for: Impact of Clinicians' Use of Electronic Knowledge Resources on Clinical and Learning Outcomes: Systematic Review and Meta-Analysis
Source: J Med Internet Res. 2019 Jul 25;21(7):e13315. doi: 10.2196/13315 (PMC6690166; doi:10.2196/13315)
Supplement: Multimedia Appendix 1 [file jmir_v21i7e13315_app1.docx]

**Appendix 1. Search strategy for review of electronic knowledge resources and point-of-care learning, 1991-2017**

With support from an experienced reference librarian, on February 14, 2017, we simultaneously searched MEDLINE, Embase, PsycINFO, and the Cochrane Library Database using Ovid’s integrated search interface. The search strategy was iteratively crafted and informed by author brainstorming of key words and the use of MEDLINE, Embase, and PsycINFO’s thesauri for the selection of appropriate controlled vocabulary terms. We also used the research teams' files and previous reviews to create and refine the search strategy, and added to the database search by examining the full bibliography of these reviews. The search was conducted using a set search approach that breaks a search into specific concepts, in this case comparative studies AND electronic knowledge resources AND clinicians, and then combines each of these concepts. We limited our search to studies published after January 1, 1991 (the year in which the World Wide Web was first described). We made no exclusions based on language.

Database(s): Embase 1988 to 2017 Week 07, PsycINFO 1806 to February Week 1 2017, EBM Reviews - Cochrane Central Register of Controlled Trials January 2017, Ovid MEDLINE(R) Epub Ahead of Print, In-Process & Other Non-Indexed Citations, Ovid MEDLINE(R) Daily and Ovid MEDLINE(R) 1946 to Present

Search Strategy:

| **#** | **Searches** | **Results** |
| --- | --- | --- |
| 1 | exp Point-of-Care Systems/ | 10567 |
| 2 | (learn* or educat* or seek* or reflect*).ti,ab,hw,kw. | 4590843 |
| 3 | 1 and 2 | 1015 |
| 4 | exp Information Seeking Behavior/ | 3467 |
| 5 | ((("work place" or workplace or "just in time" or "practice-based" or "clinical practice") adj3 learn*) or (((electronic or computer) adj1 alert*) and (educat* or learn*)) or ((reflect* or learn*) adj2 practice) or (information adj1 seek*) or (learn* adj3 ("point of care" or "point of visit*" or "clinical encounter*" or "practice encounter*" or "clinical practice")) or "information need" or "knowledge need" or "patient-directed learning").ti,ab,hw,kw. | 33072 |
| 6 | 3 or 4 or 5 | 34051 |
| 7 | (("clinical evidence" and BMJ) or ("EBM Guidelines" and Wiley) or (Isabel adj5 (diagnos* or healthcare)) or "5-minute clinical consult" or "5-minuteclinical consult" or AskMayoExpert or BestBETs or CKS or clineguide or "clinical question-answering service" or ClinicalKey or "Crtitical Appraisal Resource" or diseasedex or dynamed or "EBM Solutions" or EBMguidelines or emedicine or epocrates or "essential evidence" or "eTG complete" or "evidence matters" or "evidence-based answering service" or "First Consult" or firstconsult or "Google as a diagnostic aid" or "GP Notebook" or "grateful med" or "Harrisons Practice" or "Health Gate" or "Healthgate Clinical Guidelines" or infopoems or inforetriever or "Map of Medicine" or MAXX or "McMaster Premium Literature Service" or "McMaster-Plus" or MDChoice* or mdconsult or "md-consult" or micromedex or "national guideline clearinghouse" or "Online health knowledge resource*" or Pepid or PIER or "Quick Clinical" or RxNorm or SCAMPS or "Smart Medicine" or "Standardized Clinical Assessment and Management Plans" or "Stat Ref" or StatRef or "Technical Article Summarizer" or UpToDate or zynx or ZynxEvidence).ti,ab,hw,kw. | 7091 |
| 8 | exp rxnorm/ | 83 |
| 9 | Drug Information Services/ | 24364 |
| 10 | Information Services/ | 29024 |
| 11 | knowledge bases/ | 7768 |
| 12 | exp Textbooks as Topic/ | 65769 |
| 13 | (((bibliographic* or factual or reference or knowledge) adj3 database*) or "clinical resource*" or "diagnostic aid" or "diagnostic aids" or "five-minute clinical consult*" or "health resource*" or "information resource*" or "information service*" or "knowledge base*" or "knowledge resource*" or "knowledge support system*" or knowledgebase* or "medical database*" or "medical text" or "medical texts" or "online evidence" or "reference book*" or "text summarization*" or textbook*).ti,ab,hw,kw. | 257063 |
| 14 | Internet/ | 185542 |
| 15 | exp Computers, Handheld/ | 5147 |
| 16 | exp Cell Phones/ or exp Smartphone/ | 27471 |
| 17 | (Android or "cell phone*" or cellphone* or computer* or digital* or electronic or Google or internet or Ipad or Ipads or laptop* or microcomputer* or "Microsoft Surface" or "mobile device*" or online or "on-line" or PDA or PDAs or "personal digital assistant*" or "smart phone*" or smartphone* or tablet or tablets or web or wireless*).ti,ab,hw,kw. | 3339366 |
| 18 | or/14-17 | 3345976 |
| 19 | or/8-13 | 334640 |
| 20 | 18 and 19 | 88576 |
| 21 | 6 or 7 or 20 | 128019 |
| 22 | exp Physicians/ | 916026 |
| 23 | exp Nurse Practitioners/ | 36531 |
| 24 | exp advanced practice nurse/ | 29642 |
| 25 | exp nurse anesthetists/ | 4494 |
| 26 | exp Physician Assistants/ | 9985 |
| 27 | exp Students, Medical/ | 78565 |
| 28 | exp psychologist/ | 75395 |
| 29 | exp PSYCHOLOGISTS/ | 92890 |
| 30 | (((physician* or doctor*) adj1 (assistant* or extender*)) or "advanced practice nurse*" or Allergist* or Anaesthesiologist* or Andrologist* or Anesthesiologist* or Audiologist* or cardiologist* or clinician* or dermatologist* or doctor* or endocrinologist* or Epidemiologist* or "family practitioner*" or feldsher* or fellow or fellows or gastroenterologist* or gastrologist* or "general practitioner*" or Geneticist* or geriatrician* or gerontologist* or Gynecologist* or Hematologist* or Hepatologist* or hospitalist* or Immunologist* or "Infectious Disease Specialist*" or "Internal Medicine Specialist*" or Internist* or "medical student*" or Microbiologist* or Neonatologist* or nephrologist* or neurologist* or Neurophysiologist* or neurosurgeon* or "nurse anaesthetist*" or "nurse anesthetist*" or "nurse practitioner*" or "nurse specialist*" or obstetrician* or oncologist* or Ophthalmologist* or Orthopedist* or otolaryngologist* or pathologist* or Pediatrician* or Physiatrist* or physician* or Physiologist* or podiatrist* or practitioner* or Primatologist* or provider* or Psychiatrist* or psychologist* or Pulmonologist* or radiologist* or resident* or rheumatologist* or surgeon* or urologist*).ti,ab,hw,kw. | 3694104 |
| 31 | or/22-30 | 3773177 |
| 32 | 21 and 31 | 32726 |
| 33 | exp Treatment Outcome/ | 2196335 |
| 34 | exp "Cost Savings"/ or exp "Costs and Cost Analysis"/ | 555167 |
| 35 | exp Cost-Benefit Analysis/ | 146078 |
| 36 | exp personal satisfaction/ | 203548 |
| 37 | exp physician attitude/ | 45686 |
| 38 | exp Clinical Competence/ | 126418 |
| 39 | exp Practice Patterns, Physicians'/ | 309144 |
| 40 | exp Time Factors/ | 1151929 |
| 41 | (((needs or seek*) adj3 information) or (impact* adj3 (patient* or clinical)) or (knowledge adj3 (acquir* or aquisition*)) or (practice adj3 (pattern* or characteristic*)) or "clinical competenc*" or "clinical impact" or cost or costs or design* or economic* or implement* or outcome*).ti,ab,hw,kw. | 10122368 |
| 42 | ((((physician* or doctor*) adj1 (assistant* or extender*)) or "advanced practice nurse*" or Allergist* or Anaesthesiologist* or Andrologist* or Anesthesiologist* or Audiologist* or cardiologist* or clinician* or dermatologist* or doctor* or endocrinologist* or Epidemiologist* or "family practitioner*" or feldsher* or fellow or fellows or gastroenterologist* or gastrologist* or "general practitioner*" or Geneticist* or geriatrician* or gerontologist* or Gynecologist* or Hematologist* or Hepatologist* or hospitalist* or Immunologist* or "Infectious Disease Specialist*" or "Internal Medicine Specialist*" or Internist* or "medical student*" or Microbiologist* or Neonatologist* or nephrologist* or neurologist* or Neurophysiologist* or neurosurgeon* or "nurse anaesthetist*" or "nurse anesthetist*" or "nurse practitioner*" or "nurse specialist*" or obstetrician* or oncologist* or Ophthalmologist* or Orthopedist* or otolaryngologist* or pathologist* or Pediatrician* or Physiatrist* or physician* or Physiologist* or podiatrist* or practitioner* or Primatologist* or Psychiatrist* or psychologist* or Pulmonologist* or radiologist* or resident* or rheumatologist* or surgeon* or urologist*) adj5 (behavior* or knowledge or satisf* or skill or time or competenc*)).ti,ab,hw,kw. | 172913 |
| 43 | or/33-42 | 11443773 |
| 44 | 32 and 43 | 21518 |
| 45 | exp controlled study/ | 5679967 |
| 46 | exp Randomized Controlled Trial/ | 911991 |
| 47 | exp comparative study/ | 2731106 |
| 48 | exp intervention studies/ | 43750 |
| 49 | exp Cross-Over Studies/ | 126613 |
| 50 | exp Cohort Studies/ | 2064313 |
| 51 | exp longitudinal study/ | 331007 |
| 52 | exp retrospective study/ | 1151270 |
| 53 | exp prospective study/ | 912978 |
| 54 | exp observational study/ | 161127 |
| 55 | exp clinical trial/ | 2061411 |
| 56 | clinical study/ | 225760 |
| 57 | exp Evaluation Studies/ | 261599 |
| 58 | exp qualitative research/ | 90932 |
| 59 | exp Grounded Theory/ | 10365 |
| 60 | (((observation or observational) adj (study or survey or analysis)) or ((study or trial or random* or control*) and compar*) or (constant adj1 (comparative or comparison)) or (control* adj3 study) or (control* adj3 trial) or (grounded adj (theor* or study or studies or research or analys*)) or (intervention* adj2 study) or (intervention* adj2 trial) or (randomised adj3 study) or (randomised adj3 trial) or (randomized adj3 study) or (randomized adj3 trial) or (them* adj1 analys*) or "clinical study" or "clinical trial" or cohort* or "comparative analysis" or "comparative study" or "comparative survey" or "content analys*" or crossover or "cross-over" or "discourse analys*" or ethnograph* or "evaluation analysis" or "evaluation study" or "evaluation survey" or interview or "longitudinal analysis" or "longitudinal evaluation" or "longitudinal study" or "longitudinal survey" or "mixed method" or "narrative analys*" or phenomenol* or "pragmatic clinical trial" or "pre-post" or "qualitative analysis" or "qualitative design" or "single-group" or thematic or ((retrospective or "ex post facto") adj3 (study or survey or analysis or design)) or "prospective study" or "prospective survey" or "prospective analysis" or "prospective design").ti,ab,hw,kw,pt. | 16662090 |
| 61 | or/45-60 | 17491787 |
| 62 | 44 and 61 | 9564 |
| 63 | ((Android or "cell phone*" or cellphone* or computer* or digital* or electronic or Google or internet or Ipad or Ipads or laptop* or microcomputer* or "Microsoft Surface" or "mobile device*" or online or "on-line" or PDA or PDAs or "personal digital assistant*" or "smart phone*" or smartphone* or tablet or tablets or web or wireless*) adj3 (((bibliographic* or factual or reference or knowledge) adj3 database*) or "clinical resource*" or "diagnostic aid" or "diagnostic aids" or "five-minute clinical consult*" or "health resource*" or "information resource*" or "information service*" or "knowledge base*" or "knowledge resource*" or "knowledge support system*" or knowledgebase* or "medical database*" or "medical text" or "medical texts" or "online evidence" or "reference book*" or "text summarization*" or textbook*)).ti,ab,hw,kw. | 8123 |
| 64 | ("Clinical practice learning" or "evidence based learning" or "Information seeking" or "just in time education" or "Just in time learning" or "learning evidence based medicine" or "learning in clinical practice" or "Learning in practice" or "learning in the work place" or "learning in the workplace" or "Patient-directed learning" or "Point of care education" or "point of care information reference*" or "Point of care learning" or "Point of care reflection" or "Point of care seeking" or "Practice-based learning" or "Reflection on learning" or "work place learning" or "Workplace learning").ti,ab,hw,kw. | 14467 |
| 65 | 3 or 4 or 63 or 64 | 23422 |
| 66 | 31 and 65 | 7789 |
| 67 | 7 or 62 or 66 | 20940 |
| 68 | 67 not "conference abstract".pt. | 18666 |
| 69 | limit 68 to (editorial or erratum or letter or note or addresses or autobiography or bibliography or biography or blogs or comment or dictionary or directory or interactive tutorial or interview or lectures or legal cases or legislation or news or newspaper article or overall or patient education handout or periodical index or portraits or published erratum or video-audio media or webcasts) [Limit not valid in Embase,PsycINFO,CCTR,Ovid MEDLINE(R),Ovid MEDLINE(R) Daily Update,Ovid MEDLINE(R) In-Process,Ovid MEDLINE(R) Publisher; records were retained] | 396 |
| 70 | 68 not 69 | 18270 |
| 71 | limit 70 to yr="1991 -Current" | 17796 |
| 72 | limit 71 to yr="2014 -Current" | 4954 |
| 73 | remove duplicates from 72 | 3392 |
| 74 | limit 71 to yr="2010 -2013" | 4860 |
| 75 | remove duplicates from 74 | 3201 |
| 76 | limit 71 to yr="2005 -2009" | 3897 |
| 77 | remove duplicates from 76 | 2545 |
| 78 | 71 not (72 or 74 or 76) | 4085 |
| 79 | remove duplicates from 78 | 2624 |
| 80 | 73 or 75 or 77 or 79 | 11762 |

NOTE: From these 11,762 records we manually removed 925 additional duplicate records of the same citation, leaving 10,837 unique citations identified for screening.
