# Supplementary material for: Impact of Clinicians' Use of Electronic Knowledge Resources on Clinical and Learning Outcomes: Systematic Review and Meta-Analysis
Source: J Med Internet Res. 2019 Jul 25;21(7):e13315. doi: 10.2196/13315 (PMC6690166; doi:10.2196/13315)
Supplement: Multimedia Appendix 2 [file jmir_v21i7e13315_app2.docx]

**Supplemental Table 1. Detailed listing of all contrasts and outcomes, by study**

| Author, Year | Comparison Type | Outcomes |
| --- | --- | --- |
| Leung, 2003 (21) | Knowledge resource compared vs no intervention | Attitudes |
| Leung, 2003 (21) | Knowledge resource compared vs other resource | Attitudes |
| Schwartz, 2003 (22) | Knowledge resource compared vs other resource | Knowledge / skills |
| D’Allessandro, 2004 (23) | Knowledge resource compared vs other resource | Knowledge / skills |
| D’Allessandro, 2004 (23) | Knowledge resource compared vs other resource | Knowledge / skills |
| Alper, 2005 (24) | Knowledge resource compared vs. other resource | Knowledge / skills |
| Alper, 2005 (24) | Knowledge resource compared vs other resource | Knowledge / skills |
| Alper, 2005 (24) | Knowledge resource compared vs other resource | Knowledge / skills |
| Grad, 2005 (25) | Comparison between knowledge resources | Knowledge / skills |
| Grad, 2005 (26) | Knowledge resource compared vs no intervention | Knowledge / skills |
| Griever, 2005 (27) | Knowledge resource compared vs no intervention | Behaviors |
| Griever, 2005 (27) | Knowledge resource compared vs no intervention | Patient effects |
| Bochicchio, 2006 (28) | Knowledge resource compared vs no intervention | Knowledge / skills |
| Maviglia, 2006 (29) | Comparison between knowledge resources | Knowledge / skills |
| Maviglia, 2006 (29) | Comparison between knowledge resources | Knowledge / skills |
| Ramnarayan, 2006 (30) | Knowledge resource compared vs no intervention | Behaviors |
| Rudkin, 2006 (31) | Knowledge resource compared vs other resource | Knowledge / skills |
| Rudkin, 2006 (31) | Knowledge resource compared vs other resource | Knowledge / skills |
| Emery, 2007 (32) | Knowledge resource compared vs no intervention | Behaviors |
| Emery, 2007 (32) | Knowledge resource compared vs no intervention | Behaviors |
| Emery, 2007 (32) | Knowledge resource compared vs no intervention | Patient effects |
| King, 2007 (33) | Knowledge resource compared vs no intervention | Behaviors |
| King, 2007 (33) | Knowledge resource compared vs no intervention | Patient effects |
| Magrabi, 2007 (34) | Knowledge resource compared vs no intervention | Attitudes |
| Skeate, 2007 (35) | Knowledge resource compared vs no intervention | Knowledge / skills |
| Skeate, 2007 (35) | Knowledge resource compared vs no intervention | Knowledge / skills |
| Van Duppen, 2007 (36) | Knowledge resource compared vs other resource | Knowledge / skills |
| Van Duppen, 2007 (36) | Knowledge resource compared vs other resource | Knowledge / skills |
| Van Duppen, 2007 (36) | Comparison between knowledge resources | Knowledge / skills |
| Bonis, 2008 (37) | Knowledge resource compared vs no intervention | Patient effects |
| Hoogendam, 2008 (38) | Knowledge resource compared vs other resource | Knowledge / skills |
| Hoogendam, 2008 (38) | Knowledge resource compared vs other resource | Knowledge / skills |
| Lyman, 2008 (39) | Knowledge resource compared vs no intervention | Behaviors |
| Isaac, 2012 (40) | Knowledge resource compared vs no intervention | Patient effects |
| Isaac, 2012 (40) | Knowledge resource compared vs no intervention | Patient effects |
| Reed, 2012 (41) | Knowledge resource compared vs no intervention | Knowledge / skills |
| Reed, 2012 (41) | Comparison between knowledge resources | Knowledge / skills |
| Kuhn, 2015 (42) | Knowledge resource compared vs no intervention | Patient effects |
| Chow, 2016 (43) | Comparison between knowledge resources | Behaviors |
| Luther, 2016 (44) | Knowledge resource compared vs no intervention | Costs |
| Luther, 2016 (44) | Knowledge resource compared vs no intervention | Behaviors |
| Luther, 2016 (44) | Knowledge resource compared vs no intervention | Patient effects |
| Saparova, 2016 (45) | Comparison between knowledge resources | Knowledge / skills |
| Saparova, 2016 (45) | Comparison between knowledge resources | Knowledge / skills |
| Saparova, 2016 (45) | Comparison between knowledge resources | Knowledge / skills |
